# Gallic-Acid-Loaded PLGA Nanoparticles: A Promising Transdermal Drug Delivery System with Antioxidant and Antimicrobial Agents

**DOI:** 10.3390/ph16081090

**Published:** 2023-07-31

**Authors:** Mohammed F. Aldawsari, Faisal K. Alkholifi, Ahmed I. Foudah, Mohammed H. Alqarni, Aftab Alam, Mohamad Ayman Salkini, Sherouk Hussein Sweilam

**Affiliations:** 1 Department of Pharmaceutics, College of Pharmacy, Prince Sattam bin Abdulaziz University, Al Kharj 11942, Saudi Arabia; moh.aldawsari@psau.edu.sa; 2 Department of Pharmacology & Toxicology, College of Pharmacy, Prince Sattam bin Abdulaziz University, Al Kharj 11942, Saudi Arabia; f.alkholifi@psau.edu.sa; 3 Department of Pharmacognosy, College of Pharmacy, Prince Sattam bin Abdulaziz University, Al Kharj 11942, Saudi Arabia; a.foudah@psau.edu.sa (A.I.F.); m.alqarni@psau.edu.sa (M.H.A.); a.alam@psau.edu.sa (A.A.); s.sweilam@psau.edu.sa (S.H.S.); 4 Department of Pharmacognosy, Faculty of Pharmacy, Egyptian Russian University, Cairo-Suez Road, Badr City, Cairo-Suez Road, Cairo 11829, Egypt

**Keywords:** drug delivery system, gallic acid, transdermal administration, poly(lactic-co-glycolic acid) nanoparticles, antioxidant and antibacterial effects

## Abstract

The objective of this study was to develop an innovative gallic-acid (GA) drug delivery system that could be administered transdermally, resulting in enhanced therapeutic benefits and minimal negative consequences. The method employed involved the preparation of poly(lactic-co-glycolic acid) (PLGA) nanoparticles loaded with GA through nanoprecipitation-denoted GA@PLGANPs. The results reveal that this strategy led to perfectly spherical, homogeneous, and negatively charged particles, which are suitable for administration via skin patches or ointments. A further analysis indicates that these GA@PLGANPs exhibit remarkable antioxidant activity as well as potent antibacterial effects against a diverse range of microorganisms, making them ideal candidates for numerous applications. Additionally, it has been observed that these nanoparticles can effectively mitigate oxidative stress while also significantly inhibiting microbial growth by exerting detrimental effects on bacterial cell walls or membranes. In conclusion, on the basis of the findings presented in this study, there is strong evidence supporting the potential use of GA@PLGANPs as an effective therapy option with reduced side effects compared to conventional drug delivery methods.

## 1. Introduction

Reactive oxygen species (ROS) are reactive molecules that consist of oxygen and are integral byproducts of cellular metabolism. These innate compounds are of significant importance in regular physiological functions within an organism, serving as secondary messengers that affect various bodily processes. ROS engage with multiple signalling pathways and molecular targets across cells, modulating gene expression, protein activity, and other crucial biological activities. Therefore, these versatile compounds have emerged as pivotal contributors in a variety of biological contexts ranging from the immune response to ageing and pathological conditions [1].

The overproduction of ROS can have negative impacts on cellular wellbeing, as it may impair critical elements such as lipids, proteins, and DNA. This occurrence results in oxidative stress, which might augment the probability of developing severe ailments like cancer, diabetes, and neurodegenerative diseases. As a result, the proper control of ROS levels within cells is necessary to ensure ideal physiological functioning while avoiding any potential harm that could arise from unregulated amounts [2].

Maintaining a delicate equilibrium between ROS generation and antioxidant cellular defence mechanisms is an essential determinant that significantly influences overall cell health. This intricate balance plays a crucial role in protecting against various diseases, highlighting its immense importance within biological systems. Any disruption of this fundamental interaction may result in oxidative stress, leading to grave implications such as cancer, neurodegenerative disorders, and cardiovascular disease. Therefore, understanding and regulating ROS levels while enhancing antioxidant defences are critical determinants for preserving optimal cell function to ensure long-lasting health outcomes [3]. This can be achieved through a healthy diet rich in antioxidants, regular exercise, and avoiding exposure to environmental toxins and pollutants that can increase ROS production [4].

Among these, the gallic acid (GA) moiety (3,4,5-trihydroxybenzoic acid) moiety is essential for its antioxidant activity and has been shown to have a protective effect against ROS-induced damage [5]. GA, a polyphenolic compound with powerful antioxidant properties, is of interest to academics and specialists. Research has examined the health benefits of this chemical compound. Grapes, berries, and tea leaves contain it. GA is used as a standard in antioxidant potential studies because it is a robust free-radical scavenger with high antioxidant activity. This powerful natural therapy may reduce ROS-induced oxidative stress and maintain optimal health. The bioactive components of gallic acid may reveal even more fascinating health benefits [6]. Because of its unique qualities, it can be a powerful way to eliminate free radicals. These energetic chemicals destroy biological components and cause sickness. This chemical reduces oxidative stress and improves general health by eliminating these toxic substances [7].

GA shows potential for developing new drugs and therapeutic agents. It possesses antioxidant, antimicrobial, anti-inflammatory, and anticancer properties. Its ability to induce cell death selectively in cancer cells suggests promise for novel treatments. Continued research may unlock its full medicinal benefits [8]. In addition, GA has been observed to exhibit an impressive potential to enhance the function of antioxidant enzymes. This organic compound derived from natural sources operates via a mechanism that selectively activates pivotal proteins such as catalase and superoxide dismutase, which effectively counteract deleterious ROS. By enhancing these fundamental cellular defence systems against oxidative stressors, GA could potentially contribute significantly to maintaining optimal health and promoting general wellness [9]. Interestingly, GA has been reported to have several pharmacological properties, mainly antioxidant, anti-tyrosinase, antimicrobial, anti-inflammatory, anticancer, and neuroprotective activities [10]. In addition, it has an antibacterial property against *Escherichia coli*, *Staphylococcus aureus*, *Pseudomonas aeruginosa,* and *Klebsiella pneumonia* [11].

GA is known for its therapeutic properties, but it faces limitations in terms of bioavailability and stability. The absorption rate of the compound is restricted while rapid elimination further undermines its efficacy as a therapeutic agent. Despite these issues, researchers continue to investigate strategies to improve gallic acid’s bioavailability and stability so that its full potential can be harnessed for medicinal applications. It appears that pursuing this avenue could lead to meaningful advancements in the medical domain [12]. In-depth research is indispensable to gain a complete understanding of the safety and medicinal advantages offered by GA for human consumption. Pharmacokinetic analyses have revealed that after oral intake, this compound is quickly assimilated into the body and excreted. Hence, modifications in structure or dosage formulations might confer notable benefits in enhancing its bioavailability levels. A better understanding of these aspects will pave the way to harness the full potential of this constituent as an invaluable adjunct to various medical therapies and supplements aimed at improving global health outcomes across populations [13].

Using nanoformulation technology, hurdles that have previously impeded progress can be overcome. Utilising this cutting-edge method has led to a revolutionary advancement in harnessing the power of GA, a phytoconstituent compound renowned for its potent antioxidant capabilities. By formulating GA on a nanoscale level, it is now possible to effectively protect against free radical damage and enhance overall health outcomes across multiple domains. This field deserves further investigation and expansion to fully realise its potential benefits [14].

Nanoparticles can be used as a GA delivery system to overcome these challenges of the low bioavailability and stability of gallic acid. Nanoparticles are small particles that can improve the solubility, stability, and bioavailability of GA [15]. The encapsulation of GA in nanoparticles can protect it from degradation and increase its stability. This can enhance its effectiveness as an antioxidant and antimicrobial agent. In addition, nanoparticles can improve the solubility and bioavailability of gallic acid, allowing a more efficient delivery to target sites in the body. The use of nanoparticles can also allow for the controlled release of GA, providing sustained therapeutic effects over time. Overall, the use of nanoparticles can improve the efficacy of GA and provide a more effective means of delivering its therapeutic benefits. This can enhance its effectiveness as an antioxidant and antimicrobial agent.

To achieve this, poly(lactic-co-glycolic acid) (PLGA) nanoparticles are one of the best alternatives. PLGA is a biocompatible and biodegradable polymer that has been extensively used for drug delivery applications [16]. When GA is encapsulated within PLGA nanoparticles, several benefits can be achieved, including an enhanced stability, improved bioavailability, and controlled release for sustained therapeutic effects. This innovative approach has the potential to act as a potent antioxidant and antimicrobial agent against chronic diseases such as cancer, cardiovascular disease, and neurodegenerative disorders. In addition, it protects GA from degradation while simultaneously increasing its cellular uptake, resulting in a greater efficacy as an antioxidant agent. The slow-release mechanism of this process ensures maximum therapeutic benefit with a minimal dosing frequency, which significantly reduces the patient’s burden. In addition to these advantages, the inclusion of GA inside these particles also mitigates cytotoxicity concerns, making them much safer alternatives for administration compared to the unencapsulated forms.

The utilisation of PLGA nanoparticles containing GA as a potential treatment for diseases presents a highly promising avenue. This innovative technology offers multiple benefits, including antioxidative properties that combat free radicals and antimicrobial capabilities that target harmful microorganisms. The intricate design of these nanoparticles ensures an optimal delivery and targeted action, resulting in an improved efficacy with a reduced toxicity compared to traditional treatments. This breakthrough holds immense potential in revolutionizing the available treatment options by providing novel approaches with deeper healing benefits for various ailments.

## 2. Results

### 2.1. Percentage Encapsulation Efficiency (%EE)

The percent encapsulation efficiency (%EE) of GA@PLGANPs was carefully calculated and yielded a remarkable value of 85.073 ± 1.99%. A thorough evaluation revealed the crucial role that PLGA concentration played in determining the efficacy of drug entrapment within these nanoparticles, underscoring their potential as a novel drug delivery system for therapeutic applications. As we delved deeper into our analysis, it became increasingly clear just how significant an impact PLGA concentration had on optimizing and enhancing overall encapsulation efficacy. Results indicated that even slight variations in this key factor could have highly consequential effects on improving drug delivery through advanced nanotechnology-based approaches. These findings represent an important step forward in understanding the intricate mechanisms underlying efficient drug delivery and provide valuable insights for future research endeavours aimed at further refining targeted therapies using cutting-edge technologies like those employed with GA@PLGANPs. 

### 2.2. Particle Size and Zeta Potential

Upon conducting a thorough analysis, it was revealed that the particle size of the substance under examination measured at an impressive 336 nm with an exceedingly low PDI reading of 39.4% indicative of a high degree of homogeneity and uniformity within the sample. In addition, the zeta potential value was observed at (−) 21 mV, which provides valuable insight into the electrostatic stability and charge distribution behaviour present in these particles.

### 2.3. Morphological Study Using SEM

**Figure 1:**
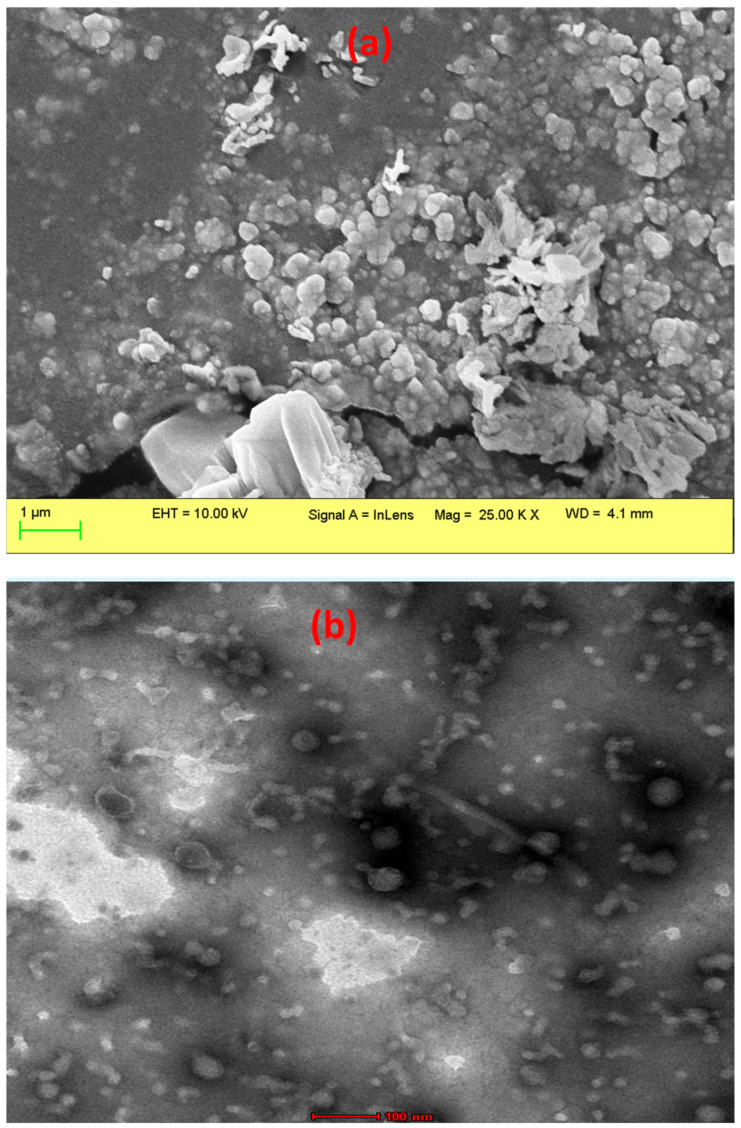
Morphological study of GA@PLGANPs observed under SEM (**a**) and TEM (**b**).

The GA@PLGANPs were thoroughly investigated to gain a more comprehensive understanding of their surface morphology. Through the use of SEM, we were able to observe the nanoparticles in great detail, utilising magnifications up to 25.00 K for optimal visualization. As shown in Figure 1a, our findings indicate that these particles possess an irregular shape and exhibit a smooth surface texture—indicative of homogenous composition and meticulous synthesis procedures. In our study, the successful formation of nanoparticles with the desired morphology was confirmed through the TEM analysis as shown in Figure 1b. The TEM images revealed the presence of spherical particles with an approximate size of 100 nm, which is significant and meets the desired range for optimal function. The uniform distribution and consistent structure indicated by the spherical shape of the particles are advantageous for their intended applications. Overall, the SEM and TEM analysis results validate the effectiveness of the nanoprecipitation method employed in this study, demonstrating the successful synthesis of spherical nanoparticles with the desired size.

### 2.4. In Vitro Drug Release Study

**Figure 2:**
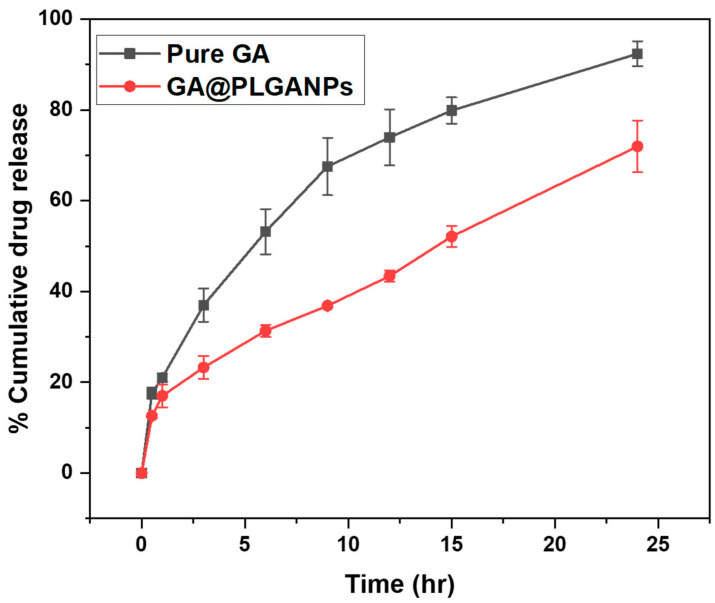
In vitro drug release profile of GA@PLGANPs in PBS at pH 7.4.

The study involved conducting an in vitro analysis of the release of GA from two sources: PLGA nanoparticles and pure GA. The objective was to evaluate the ability of these nanoparticles to regulate and extend the duration of GA release, as illustrated in Figure 2. This investigation aimed at providing a more comprehensive understanding on how controlled drug delivery systems can potentially enhance therapeutic efficacy by sustaining optimal concentrations over prolonged periods. The study’s results illuminated the intricate behaviour of GA@PLGANPs as a drug delivery system. It was discovered that PLGA gradually underwent degradation, which led to a consistent and sustained release of drugs over time. The pattern observed in the drug release exhibited two distinct phases, an initial burst phase followed by an extended sustained-release phase lasting for up to 24 h. The results of the study showed that GA@PLGANPs had a drug release amount of 31.34 ± 4.45% after 6 h, which significantly increased to an impressive rate of 71.99 ± 4.35% by the end of 24 h, suggesting significant potential for optimal therapeutic outcomes when used in clinical settings. On the other hand, pure GA exhibited a higher initial drug release with levels at around 53.18 ± 4.97% within 6 h and increasing exponentially over time to reach a maximum level of about 92.54 ± 2.72% at the 24-h mark. These findings reveal promising implications for future research on developing novel drugs that can target specific cell types or organs while also being effective in treating various diseases effectively with minimal associated side effects and radiation therapy interventions, ultimately improving patient outcomes overall.

Further, we performed the drug-release kinetics, which is a crucial area in pharmaceutical research that involves investigating the time-dependent behaviour of drugs after administration s. They encompass an array of techniques and methodologies aimed at understanding various aspects, such as the rate and extent of drug dissolution, absorption, distribution, metabolism, and excretion. Through this study, researchers can obtain valuable insights into how to optimize drug formulations for better therapeutic outcomes while minimizing adverse effects on patients.

**Figure 3:**
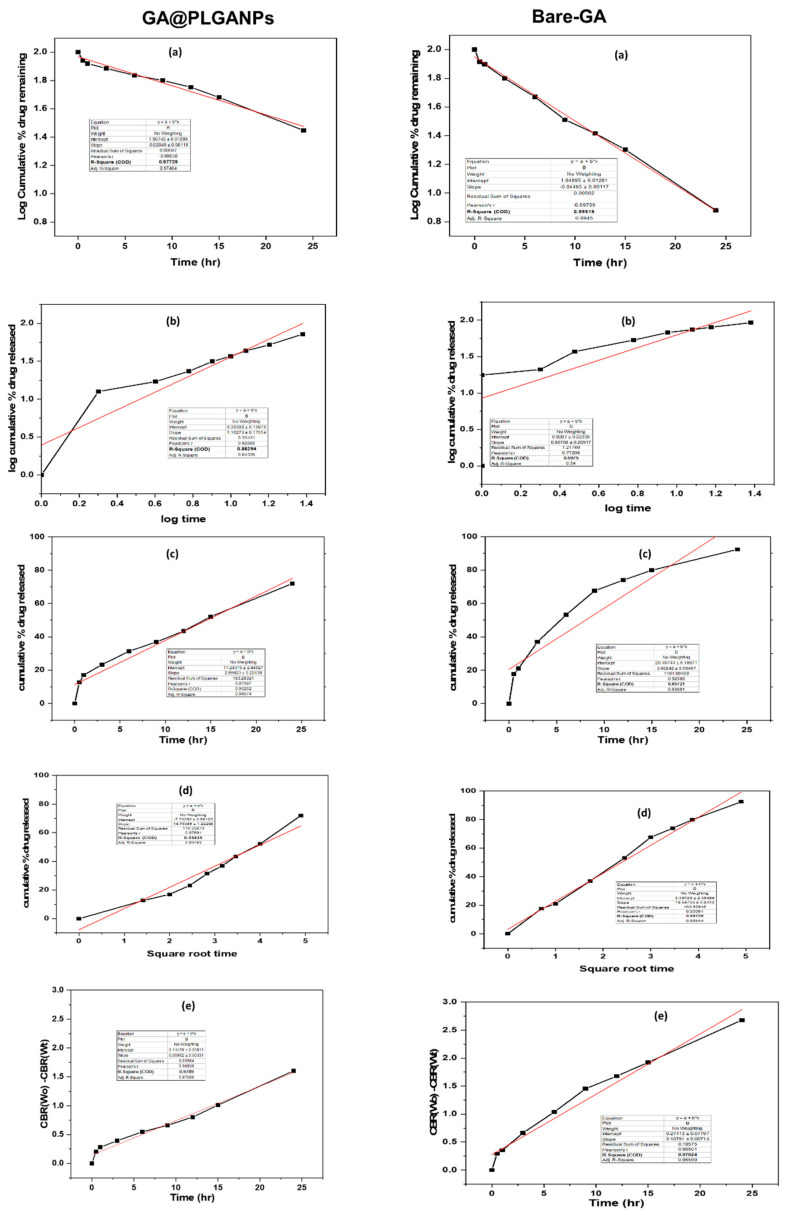
Drug-release kinetics of GA@PLGANPs and pure GA by using a different kinetics model. (**a**) First-order; (**b**) Korsmeyer–Peppas; (**c**) zero-order; (**d**) Higuchi; and (**e**) Hixson–Crowell models.

Upon conducting a thorough analysis, it was revealed that the drug-release kinetics exhibit exceptional characteristics with an impressive r^2^ value of 0.978. Notably, among all other models considered in this study (as illustrated in Figure 3 and Table 1), the Higuchi model exhibited the highest level of performance. This finding speaks volumes about its potential for future applications in drug delivery systems. Moreover, when examining pure GA, we observed an equally remarkable r^2^ value of 0.978 but with first-order kinetics as opposed to the Higuchi model’s superiority overall. The drug release of GA is governed by first-order kinetics, where changes in the rate of release or remaining concentration can affect how drugs are delivered and metabolized within living organisms.

### 2.5. In Vitro Antioxidant Activity

**Figure 4:**
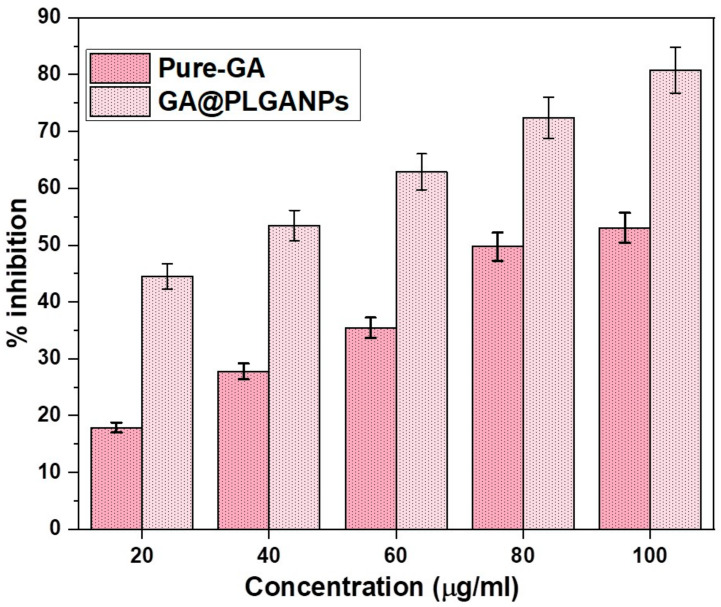
Comparative in vitro % inhibition of GA@PLGANPs and pure GA in DPPH.

The results of the study indicate a significant difference in antiradical activity between GA@PLGANPs and pure GA, with the former displaying a superior performance. Specifically, at concentrations ranging from 20 to 100 μL, both substances were found to exhibit a considerable inhibition of DPPH radicals, as demonstrated in Figure 4. However, a closer analysis reveals that the IC50 value for GA@PLGANPs was substantially lower than that of pure GA (32.04 μg/mL vs. 88.69 μg/mL). These findings suggest that incorporation into PLGA nanoparticles enhances the antioxidant efficacy of GA by facilitating its more effective delivery and bioavailability within biological systems. Overall, this research provides valuable insights into potential strategies for improving therapeutic outcomes through targeted drug encapsulation techniques.

### 2.6. In Vitro Antimicrobial Activity

The primary objective of this study was to conduct a comprehensive assessment aimed at determining the minimum inhibitory concentration (MIC) levels of GA, GA@PLGANPs, and PLGANPs in relation to their effectiveness against *S. aureus* (ATCC 25923). Interestingly, results revealed that the antimicrobial potency exhibited by GA@PLGANPs considerably surpassed that of pure GA. It is worth noting that statistical analyses demonstrated significant dissimilarities between pure GA and GA@PLGANPs regarding their ability to inhibit the growth of *S. aureus* with a *p*-value less than 0.05.

The study explored the potential of pure GA and GA@PLGANPs to augment antibacterial efficacy against *S. aureus*, revealing a complex interplay of factors contributing towards this enhancement. These included the occlusive nature of these agents, specific drug–carrier interactions, as well as their ability to establish close contact with bacterial layers due to their small size. Notably, vancomycin, a standard antibiotic, displayed an MIC value that was surpassed by both GA@PLGANPs (15.22 ± 1.85) and GA (79.33 ± 3.78). Overall, these findings suggest that exploiting synergistic effects between naturally occurring compounds and nanoparticles may hold significant promise in developing novel therapeutic strategies against microbial infections caused by resistant strains like *S. aureus*, as given in Table 2.

### 2.7. In Vitro Cell Viability Assay

**Figure 5:**
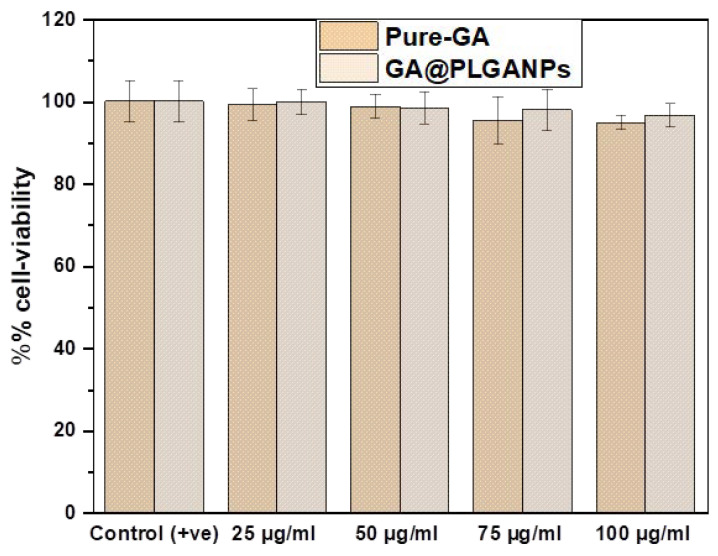
Comparative in vitro % cell viability of GA@PLGANPs and pure GA in HaCaT.

In this study, the cytotoxicity of GA@PLGANPs and GA was evaluated using normal cell lines HaCaT in the concentrations ranging from 25 to 100 μL. The results revealed that both GA@PLGANPs and GA had no significant effect on the viability of more than 75% of the cells tested, as demonstrated in Figure 5. This finding suggests that these new synthetic nanoformulations have an excellent biocompatibility with a minimal toxicity towards human skin keratinocytes. Furthermore, these outcomes indicate promising potential for utilising GA@PLGANPs and GA in various in vitro applications. It can be inferred from these findings that this novel approach to designing nanoparticles is not only efficient but also safe for use within biological systems due to its low cytotoxicity profile. Furthermore, these results strongly support an assertion regarding low toxicity levels and an outstanding biocompatibility between GA@PLGANPs and GA within physiological systems, thereby implying their potential effectiveness in various vitreous applications such as in vitro testing environments.

## 3. Discussion

GA (3, 4, 5-trihydroxybenzoic acid) is an endogenous product in plants that can be used as a powerful antioxidant and antimicrobial agent. When formulated into poly(lactic-co-glycolic acid) nanoparticles, it shows promising potential for various applications in the fields of medicine, food preservation, and cosmetics. The encapsulation of GA within PLGA nanoparticles enables the controlled release of the active ingredient over time. This sustained delivery mechanism allows for an optimized efficacy and reduced toxicity compared to traditional methods of treatment. Furthermore, nanoencapsulation enhances the bioavailability and stability of GA during storage or transportation. With its strong antioxidant properties, GA has been shown to have potential health benefits such as reducing inflammation and oxidative stress, leading to the prevention of chronic diseases like cancer or Alzheimer’s disease, among other effects when administered through nanoparticle vehicles.

The encapsulation efficiency of GA in PLGA nanoparticles was meticulously determined through a rigorous calculation process, resulting in an exceptional and noteworthy value of 85.073 ± 1.99%. Such a high percentage highlights the excellent capability of a PLGA nanoparticle to efficiently encapsulate GA with a great accuracy and precision, reflecting the quality and superiority of this innovative technology.

The analysis showed a particle size of 336 nm and PDI reading of 0.03, indicating a high consistency and uniformity in the substance’s composition. Examining the zeta potential value revealed insights into electrostatic stability and charge distribution behaviour—important for evaluating particle efficacy. A zeta potential at (−) 21 mV confirms advancements in nanoparticle fabrication strategies for better integration into technological advancements with positive outcomes.

Extensive research has been carried out on the GA@PLGANPs in order to obtain a more elaborate understanding of their surface morphology. Our investigative findings, as displayed in Figure 1a,b, revealed that these nanoparticles possess an intricate irregular shape and display a supremely smooth surface texture, which strongly indicate precise composition and meticulous formulation techniques employed during synthesis procedures.

Upon analysing the results of the study, it is evident that GA@PLGANPs exhibit significant promise as potential carriers for drug-release applications in clinical settings. It was observed that after 6 h, their drug release amount stood at 31.34 ± 4.45%, which then experienced an impressive increase to a rate of 71.99 ± 4.35% by the end of 24 h, providing key insights into their prolonged and sustained effects on therapeutic outcomes. Upon a further analysis, it can be observed that the pure GA had a significantly greater drug release as compared to other samples during the initial 6 h. This was evidenced by remarkably high levels amounting to about 53.18 ± 4. The initial rate of drug diffusion was notably high, with levels measuring at approximately 53.18 ± 4.97% within 6 h after administration. As time progressed, this release continued to increase at an exponential pace and ultimately reached its peak level at around 24 h post-administration, measuring at about 92.54 ± 2.72%. This finding suggests that the lack of a coating or protective layer on GA greatly influences its performance as a drug delivery system by enabling rapid dispersion and bioavailability in vivo.

The GA@PLGANPs follow the Higuchi model, which represents drug release from various delivery systems over time. This suggests that their drug delivery mechanism is based on the sustained and controlled release of therapeutic agents, rather than a sudden burst of medication all at once. Further research may be necessary to fully understand the implications and potential applications of this finding in medicine.

Upon conducting a more comprehensive analysis, it was uncovered that the GA@PLGANPs possess exceptional levels of antioxidant activity. Not only that, but they also exert potent antibacterial effects against a broad spectrum of microorganisms. It is evident from these findings that this particular type of nanoparticle holds immense potential for utilisation in various applications due to its diverse and remarkable properties.

Upon conducting the necessary tests, it was discovered that neither GA@PLGANPs nor GA had a profound impact on the viability of more than 75% of the cells tested. It can be inferred that these compounds may have an insignificant effect on cellular activity.

## 4. Materials and Methods

### 4.1. Materials

The GA (98%) was procured from Alfa Aesar (Kandel, Germany). Poly (lactic-co-glycolic acid), PLGA, Poloxamer 188, 2,2-Diphenyl-1-picrylhydrazyl (DPPH), an MTT reagent, and vancomycin were purchased from Sigma-Aldrich. All other chemicals used were of an analytical grade and did not require further purification before use and obtained from the Department of Pharmacognosy, Prince Sattam bin Abdulaziz University (PSAU), Al-Kharj. The double-filtered deionized (DI) water was used to prepare all solutions. *Staphylococcus aureus* (ATCC 25923) was collected from the Department of Pharmaceutics, College of Pharmacy, PSAU, Al-Kharj. An HaCaT cell line was obtained as a gift from Dr. Mohammad Raish, the Department of Pharmaceutics, King Saud University (Riyadh, Saudi Arabia).

#### 4.1.1. Preparation of GA-Loaded PLGA Nanoparticles

In our study, we used the nanoprecipitation method, known for its high effectiveness, to prepare GA@PLGANPs. Initially, 50 mg of a PLGA polymer was dissolved in acetone, followed by the precise addition of GA (20 mg) to the PLGA/acetone solution. To ensure stability, a separate aqueous phase consisting of surfactant Poloxamer 188 was prepared. The organic phase was then injected into the aqueous phase (15 mL) using a 21 G needle at an injection rate of 2.5 mL/s, under continuous magnetic stirring at room temperature until the complete evaporation of the organic solvent occurred. In addition, drug-free nanoparticles were prepared using the same procedure, excluding the addition of the drug. All samples were prepared in duplicate for a further analysis [17].

#### 4.1.2. Percentage Encapsulation Efficiency (%EE)

The percentage of encapsulation efficiency is a key factor in determining the effectiveness of these nanoparticles, referring to the percentage of GA successfully incorporated into the formulation during development. To optimise the results, we carefully adjust factors such as polymer concentration and processing parameters while considering physicochemical properties that may affect the % EE and overall efficacy after administration in various biological environments. The %EE of GA nanoparticles was calculated adopting the following formula [18]:

(1) $$\mathrm{Drug}\;\%\;\mathrm{EE}:\;\frac{\mathrm{Initial}\;\mathrm{drug}-\mathrm{Free}\;\mathrm{drug}\;\mathrm{after}\;\mathrm{encapsulation}}{\mathrm{Initial}\;\mathrm{drug}}\times 100$$

#### 4.1.3. Particle Size and Zeta Potential

In our study, we utilised an Anton Paar Dynamic Light Scattering instrument, USA, to determine the particle size and zeta potential of GA@PLGANPs. To obtain a consistent dispersion of the NPs, they were initially diluted with DI water. Subsequently, DLS techniques were employed to measure the particle size distribution, while electrophoretic mobility measurements were used to evaluate the surface charge distribution. Throughout this experimentation process, standardised techniques were consistently applied to ensure reliable and optimal results [19,20].

#### 4.1.4. Morphological Study Using SEM and TEM

In our study, a comprehensive morphological analysis of GA@PLGANPs was conducted using scanning electron microscopy (SEM). For this purpose, a suspension of GA@PLGANPs was prepared. To prepare samples for the SEM analysis, a small quantity of the particle suspension was placed on copper tape and left to dry overnight. Subsequently, the sample stub was coated with platinum using an Auto Fine Platinum Coater for a duration of 50 s. The same procedure was followed for the analysis of dry powder samples, with the exception that double-sided sticky tape was used instead of copper tape and that no drying time was required before coating. Samples were observed using a JEOL JSM-6700 F microscope in Japan [20]. Furthermore, to analyse the morphology of GA@PLGANPs, a Transmission Electron Microscopy (TEM) analysis was conducted using a JEOL-2000 Ex II TEM instrument located in Akishima, Japan. To prepare the samples for TEM imaging, a carbon-coated copper grid was utilised. After a drop of GA@PLGANPs was applied onto the grid, any excess sample was carefully removed using filter paper. Subsequently, a drop of 2% PTA (*w*/*v*) (phosphotungstic acid solution) was added to the carbon-coated copper grid and left undisturbed for a duration of 2 min. Subsequently, the excess staining agent was removed using filter paper and the sample was allowed to air dry before being examined using TEM [21].

#### 4.1.5. In Vitro Drug Release Study

In our study, we conducted an in vitro drug-release experiment to assess GA release. The experiment involved using the dialysis bag diffusion technique with a molecular weight cut-off of 12,000 Daltons. We placed the GA-loaded samples in a dialysis bag and immersed them in 200 mL of phosphate-buffered saline (PBS) with a pH of 7.4. The temperature of the medium was maintained at 37 ± 0.5 °C and we ensured constant stirring at 75 rpm using a magnetic stirrer. At predetermined time intervals, we withdrew multiple samples from the receptor compartment. To maintain consistency and accuracy, we promptly replaced the withdrawn samples with a fresh medium for continuous testing. The concentration of GA in the samples was determined with a meticulous analysis using UV spectrophotometry at 274 nm [22,23].

To gain a comprehensive understanding of drug-release behaviour, we conducted a comprehensive evaluation utilising multiple models, namely the first-order, Korsmeyer–Peppas, zero-order, Higuchi, and Hixson–Crowell models. By meticulously analysing various factors and parameters associated with each model, we were able to extract valuable insights into the kinetics of drug release over time. This thorough analysis provided us with profound conclusions that would have remained elusive had we only skimmed the surface.

#### 4.1.6. In Vitro Antioxidant Activity

The DPPH scavenging activity of GA and GA@PLGANP was determined using a well-established method published by Cho et al. [24], with slight modifications to enhance precision and accuracy. In this method, a solution containing 1.5 × 10^−4^ M of DPPH (100 μL) was mixed with GA and GA@PLGANP (100 μL) as well as without it, after which the mixture was subjected to an incubation process at room temperature for precisely 30 min to ensure complete reaction kinetics were achieved. The absorbance levels of each sample in the mixture were then carefully measured at a wavelength of 517 nm using high-precision microplate readers that ensured that accurate results were obtained every time. By employing this procedure, we gained in-depth insights into the influence of various variables on our experimental outcomes, thereby providing valuable information for optimising our approach in future studies, if necessary, and achieving a better understanding of the behaviour of these systems.

#### 4.1.7. In Vitro Antimicrobial Activity

In this study, the bacterial strain employed was *S. aureus* (ATCC 25923), a well-known pathogen renowned for its virulence and resistance to various antibiotics. To maintain consistency, the turbidity of the bacterial suspension was adjusted to a standardised McFarland unit of 0.5, indicating a suitable concentration of cells per unit volume. Subsequently, for experimental purposes, the inoculum size was further diluted with a sterilised nutrient broth and Sabouraud dextrose broth in a 1:10 ratio. This dilution procedure adhered to the protocol outlined by Yousef and Danial, who are regarded as authority on microbial culturing techniques. Consequently, our final inoculum contained approximately 10^8^ colony-forming units per millilitre (CFU/mL). Such high levels of bacterial concentration were necessary to ensure precise measurements and reliable results when investigating antimicrobial properties and other pertinent characteristics associated with the biology of *S. aureus* [25].

The MICs of GA, GA@PLGANPs, and PLGANPs were determined against the *S. aureus* strain using broth microdilution with two-fold serial dilution in the nutrient broth medium and Sabouraud dextrose broth. Sterile microtiter plates were used to determine the MIC values. To prepare the experimental conditions for studying the interaction between GA, GA@PLGANPs, and PLGANPs, thorough measures were taken.

In this study, we accurately prepared stock solutions for each component, focussing on achieving complete solubility at a concentration of 1 mg/mL in water. This was crucial to ensure reliable and consistent results during the experimentation. We distributed 100 µL samples of the nutritional broth and dextrose broth into wells 1 through 10, ensuring precise measurements and an even distribution among all wells. This created a controlled environment necessary to effectively test the interactions of these compounds. From well 1, we started the experiment with a mixture of GA, GA @ PLGANP, and PLGANP in a volume of 100 µL. Subsequently, serial dilution was performed from well 1 to well 10, with 100 µL being removed from the last well. We evenly distributed a bacterial suspension (also 100 µL) across all wells, covering the dilution intervals from the initial to the final wells (numbered one through ten, respectively). To observe growth, we added bacteria (100 µL) to well 11 and a sterile broth (100 µL) as a positive control. As negative controls for sterility, we used the nutrient broth and Sabouraud’s dextrose broth in well 12. The plate was then incubated at the optimal temperature of 37 °C for 1 day. After incubation, we used an ELISA reader (Erba) to measure the absorbance at 640 nm in each well, ensuring accuracy by performing all tests in triplicates.

#### 4.1.8. In Vitro Cell Viability Assay

To assess cell viability in our study, we used the MTT assay, a commonly used method. Our analysis involved the utilisation of GA and two types of nanoparticles, namely GA@PLGANPs and PLGANPs, in a concentration ranging from 25 to 100 µg/μL. These substances were introduced into HaCaT cells for comprehensive evaluation, with the aim of gaining a deeper understanding of their impact on cell health. The technique used provided valuable insights into the effects of these compounds on cell viability [26,27]. The HaCaT cells utilised in the experiment were sourced from King Saud University, Saudi Arabia. Subsequently, careful and precise cell culturing was performed using an enhanced variant of a Dulbecco Eagle medium (DMEM) supplemented with 10% heat-activated foetal bovine serum to promote their growth and proliferation. The entire procedure adhered to strict environmental controls, including a humidified incubator maintaining a stable temperature of 37 °C and a CO_2_ concentration of 5%, ensuring optimal cellular development. To assess the viability of the cancer cells, the MTT assay was conducted.

In order to carry out this experiment, 1 × 10^4^ HaCaT cells were suspended in 100 μL of the solution and placed in separate wells within a 96-well plate. Following a period of incubation lasting for approximately 1 day, varying concentrations of GA, GA@PLGANPs, and PLGANPs were applied to each respective well for an additional duration of another full day. Afterward, the MTT (20 μL) solution was incorporated into every culture well before being allowed to develop over the course of 4 h under specific conditions designed to achieve optimal results. Upon the completion of the incubation period, the medium containing MTT was carefully removed and cells were subsequently treated with dimethyl sulfoxide (DMSO), a potent solvent. Finally, to determine cell viability, using a microculture plate reader set at a 595 nm wavelength, absorbance was measured and the subsequent determination of differentials was expressed as a percentage relative to control values [28].

$$\mathrm{Cell}\;\mathrm{viability}=\frac{\left(A595\;\mathrm{nm}\;\mathrm{treated}\;\mathrm{cells}\right)}{\left(A595\;\mathrm{nm}\;\mathrm{untreated}\;\mathrm{cells}\right)}\times 100$$

## 5. Conclusions

The present study delves into the potential applications of GA@PLGANPs, exploring both topical and oral delivery methods. Through careful experimentation utilising a nanoprecipitation technique, the researchers effectively encapsulated GA within these minute particles with remarkable results as an antioxidant and antimicrobial agent. The unique design and composition of these nanoparticles enable them to surpass biological barriers effortlessly while achieving a heightened therapeutic efficacy through precise targeting. These GA@PLGANPs are highly monodispersed in nature, with a spherical shape and diameter measuring at 336 nm. In comparison to their conventional counterparts, they possess stronger antioxidant properties along with superior antibacterial effects, making them excellent biomedical choices requiring robust antibacterial action.

Furthermore, it is essential to note that these particular GA agents hold significant promise for various fields such as pharmaceuticals or biology research studies due to their distinct attributes providing potent therapeutic actions towards specific processes. All in all, this preparation based on nanoparticle materials marks substantial advancements regarding innovation in targeted drug delivery.

## Figures and Tables

**Table 1:** Drug-release kinetics of GA@PLGANPs.

Formulation	First-Order	Korsmeyer–Peppas	Zero-Order	Higuchi Model	Hixson–Crowell Model
GA@PLGANPs	0.977	0.862	0.952	0.954	0.978
Pure GA	0.995	0.597	0.857	0.987	0.970

**Table 2:** Comparative in vitro antibacterial activity of vancomycin, GA@PLGANPs, and PLGANPs against *S. aureus* (ATCC 25923) bacteria.

Compound	MIC (µg/mL)
Vancomycin	3.656 ± 0.53
GA@PLGANPs	15.22 ± 1.85
GA	79.33 ± 3.78
PLGANPs	NA

## Data Availability

Data is contained within the article.
